# Second Trimester Ultrasound Diagnosis of External Hydrocephalus in Two Fetuses with Noonan Syndrome—Case Report Series

**DOI:** 10.3390/jcm14113973

**Published:** 2025-06-04

**Authors:** Tibor Elekes, Aniko Ladanyi, Eva Pap, Janos Szabo, Anett Illes, Nora Gullai, Szabolcs Varbiro

**Affiliations:** 1 Department of Obstetrics and Gynecology, University of Szeged, H-6725 Szeged, Hungary; ladanyia@medical.hu (A.L.); pap.eva.melinda@med.u-szeged.hu (E.P.); szajna777@gmail.com (J.S.); gullai.nora@gmail.com (N.G.); 2 Department of Internal Medicine and Oncology, Semmelweis University, H-1082 Budapest, Hungary; barkaszine.dr.illes.anett@semmelweis.hu

**Keywords:** Noonan syndrome, external hydrocephalus, subarachnoid space

## Abstract

**Background:** Noonan syndrome (NS) is a relatively common RASopathy that can be associated with a variety of phenotypic and genotypic variations and potential long-term health consequences. Its most described prenatal ultrasound features in the first trimester are thickened nuchal translucency (NT) and dilated jugular sacs; while heart defects, polyhydramnios and facial dysmorphisms are its known manifestations in the second and third trimesters. **Methods:** We present two cases of NS with the prenatal ultrasound diagnosis of external hydrocephalus (EH) in the second trimester. **Results:** Case 1 had a normal first trimester scan and showed mild polyhydramnios, an echogenic intracardiac focus (EIF) in the left ventricle and pyelectasis in the second trimester in association with the EH. The whole exome sequencing (WES) confirmed a pathogenic variant in the *SOS1* gene. Case 2 showed increased NT, agenesis of the ductus venosus (DV), single umbilical artery (SUA), an EIF in the right ventricle and an abnormal prefrontal space ratio (PSFR). By the 19th gestational week, EH appeared. The ambient and quadrigeminal cisterns were also slightly widened. The WES revealed a *PTPN11* gene variant. **Conclusions:** The most reported sonographic features of NS are either non-specific or difficult to integrate into routine screening, requiring substantial experience. In our two cases, we detected EH in the second trimester, which is rarely described as a prenatal ultrasound diagnosis. To our current knowledge, this is the first case reported of EH in NS caused by an *SOS1* gene variant and these are the first cases reported with the prenatal sonographic diagnosis of EH in NS.

## 1. Introduction

Noonan syndrome is a relatively common RASopathy associated with a variety of phenotypic and genotypic features. Its prevalence is estimated to be 1:1000–2500, but in mild cases might be higher [1,2] and, therefore, more common than Edwards or Patau syndrome [3]. It is named after Jacqueline Noonan, an American pediatric cardiologist who published a paper in 1963 describing nine patients with pulmonary valve stenosis and various other coexisting conditions [4].

NS is mainly inherited in an autosomal dominant manner and in about 50% of the cases due to a variant in the *PTPN11* gene (176876). Other known gene variations that lead to NS are *NS3* (609942), *KRAS* (190070); *NS4* (610733), *SOS1* (182530); *NS5* (611553), *RAF1* (164760); *NS6* (613224), *NRAS* (164790); *NS7* (613706), *BRAF* (164757); *NS8* (615355), *RIT1* (609591); *NS9* (616559), *SOS2* (601247); *NS10* (616564), *LZTR1* (600574); *NS11* (618499), *MRAS* (608435), *NS12* (618624), *RRAS2* (600098), *NS13* (619087) and *MAPK1* (176948), while the autosomal recessive form of Noonan syndrome is caused by *NS2* (605275), *LZTR1* (600574), *NS14* (619745) and *SPRED2* (609292) [5,6,7].

Noonan syndrome is characterized by typical facial dysmorphism, skeletal anomalies, webbed neck and congenital heart defects [1].

Following Down syndrome, Noonan syndrome is the second most common genetic disorder associated with heart defects, typically with pulmonary stenosis and hypertrophic cardiomyopathy [8].

The prenatal diagnosis of Noonan syndrome is often challenging due to variability in the phenotypic presentation and occasionally mild and non-specific signs [9,10]. In NS, the most frequently reported prenatal ultrasound findings are increased NT, cystic hygroma, dilated jugular lymphatic sacs, thickened nuchal fold, cardiac defects, kidney malformations and polyhydramnios [9,11,12,13].

In those cases in which the fetus does not show the typical signs associated with Noonan syndrome, the detection rate remains low and, due to the often non-specific findings, the diagnosis might be delayed or missed even in postnatal life [9,14].

External hydrocephalus is defined as enlarged subarachnoid spaces detected on imaging modalities with normal or mildly dilated ventricles [15]. In the presented cases, the authors used the depth of the Sylvian fissure to determine the width of the subarachnoid spaces, based on the normal value database published by Alonso et al. [16].

In this paper, we report two cases of prenatally confirmed Noonan syndrome showing the rare sonographic finding of external hydrocephalus in the second trimester.

## 2. Case Report Series

We present two cases of Noonan syndrome with external hydrocephalus in the second trimester in the presence of the variations of the *SOS1* and *PTPN11* genes. To our current knowledge, this is the first paper describing the association of prenatal external hydrocephalus in Noonan syndrome caused by a variation of the *SOS1* gene, and these are the first cases reported with a second trimester sonographic diagnosis of EH in NS. This case report is structured according to the CARE guidelines. We defined external hydrocephalus on the level of Sylvian fissure: measure over 95 percentile was defined as EH.

### 2.1. Case 1

#### 2.1.1. Patient Information and Obstetric History

The 46-year-old pregnant woman first presented for the first trimester screening at the gestational age of 12 weeks and 5 days. The mode of conception was IVF from oocyte donation (donor is under 25 years of age). She previously had a missed abortion at the 7th gestational week.

She had a medical history of antiphospholipid syndrome, hypothyroidism and insulin resistance, her regular medications were metformin, levothyroxine, liothyronine, progesterone, escitalopram in combination with clonazepam, and enoxaparin. No family history of genetic disease was known. Body mass index (BMI) was in the normal range.

#### 2.1.2. Ultrasound Findings

The first trimester extended ultrasound scan (NT, nasal bone, DV, tricuspid valve regurgitation, early cardiac and intracranial scan) revealed no anomalies. The thickness of the nuchal translucency was 2.4 mm, representing 85 percentile based on the measured CRL of 69.9 mm, which was 3 days greater than the calculated gestational age according to the IVF.

**Figure 1:**
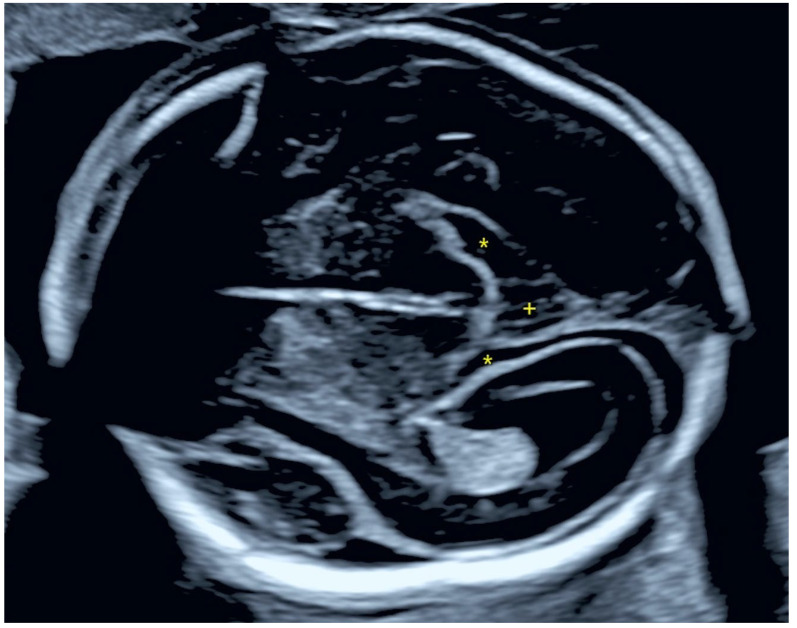
The enlarged ambient cisterns (*) and quadrigeminal cistern (+) at the 20th gestational week.

**Figure 2:**
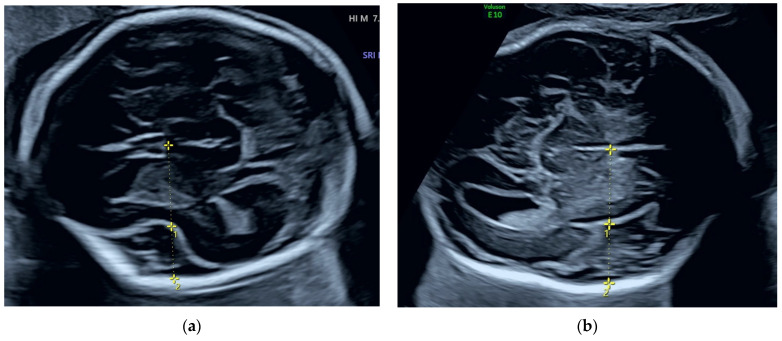
Measurements of the Sylvian fissure. Measurements marked with 1 indicate the insular depth, measurements marked with 2 indicate the Sylvian fissure depth. (**a**) The depth of the Sylvian fissure was measured 10.0 mm at the 20th week; (**b**) and 17.4 mm at the 27th week. Both measurements exceeded the 95th percentile according to the database published by Alonso et al. in 2010 [16].

In the second trimester, a mild polyhydramnios was found (deepest vertical pocket: 9.3 cm, amniotic fluid index: 21 cm). BPD (52.2 mm) and HC (191.1 mm) were above the 90th percentile, all other biometric data were in the normal range. The diameter of the posterior horn at the atrium of the lateral ventricle was 9 mm. The ambient and quadrigeminal cisterns appeared slightly dilated (Figure 1), and the Sylvian fissure measured 10.5 mm, which was above the 95th percentile (Figure 2a). In addition, a mild hypertelorism (outer-to-outer distance: 35 mm, inner-to-inner distance: 16 mm), an echogenic intracardiac focus (EIF) in the left ventricle and mild bilateral pyelectasis were detected. An echocardiogram was performed by a pediatric cardiologist, which revealed no cardiac anomalies.

**Figure 3:**
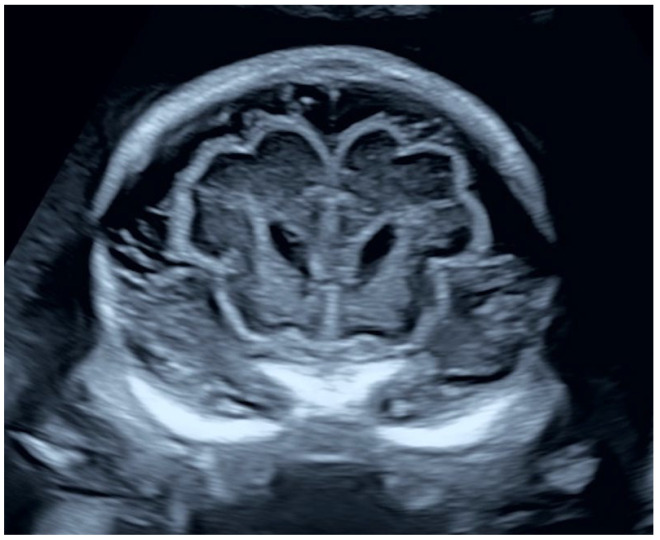
Coronal transcaudate plane in the 27th gestational week. The dilation of the subarachnoid spaces became obvious.

Fetal ultrasound follow-ups were performed at the 27th, 31st and 34th gestational week. Mild-to-moderate polyhydramnios was confirmed throughout the pregnancy. BPD and HC increased steadily, with both above the 99th percentile by the 27th gestational week. The ambient and quadrigeminal cisterns remained enlarged and the subarachnoid spaces widened. At the 27th gestational week, the depth of the Sylvian fissure measured 17.4 mm, which is high above the 95th percentile (Figure 2b) and the external hydrocephalus became evident also in the cranial views (Figure 3).

AC was measured at the 98th percentile at the 27th week, with an estimated fetal weight at the 97.9th percentile. These biometric data were maintained throughout the pregnancy. A mild dilatation of the right kidney remained with a diameter of 8 mm measured at the 34th week of pregnancy, while the width of the right kidney pelvis was normalized.

#### 2.1.3. Genetic Studies

A cf-DNA test was performed in the first trimester with normal results. An amniocentesis was performed following the second trimester scan. No chromosomal anomalies were confirmed by G-band analysis of the amniotic cell culture (46XY). The array CGH confirmed a 2 Kb heterozygous microdeletion affecting the *RAB6C* (OMIM: 612909) and *SMPD4* (OMIM: 610457) genes. The following whole exome sequencing ruled out any compound heterozygosity and revealed a heterozygous pathogenic variation in the *SOS1* gene (NM_005633.4:c.1644T>A, p.Ser548Arg) that confirmed Noonan syndrome 4 (Phenotype MIM number: 610733, location: 2p22.1, Gene/Locus MIM number: 182530).

#### 2.1.4. Perinatal and Postnatal Outcome

After detailed genetic counselling, the pregnant woman decided to proceed with the pregnancy. The baby was born by caesarean section in the 38th gestational week, with a birth weight of 3530 g, Apgar 9/10. The routine neonatal screening tests for vision and hearing were normal.

The newborn underwent regular cardiological follow-up, which revealed mild supravalvular pulmonary stenosis with no progression during the follow-up period to date. The last check-up was at age 2, requiring no intervention and annual follow-ups.

A cranial ultrasound at 7 weeks of age did not report any anomalies. The neurodevelopmental examination revealed minimal whole-body hypotonia and moderately delayed psychomotor development. Complex (motor, mental, speech) rehabilitation was recommended with a follow-up examination in 1 year.

### 2.2. Case 2

#### 2.2.1. Patient Information and Obstetric History

The 32-year-old pregnant woman was referred to our clinic with her spontaneously conceived second pregnancy. She had a previous normal vaginal delivery at the 39th gestational week following an uneventful pregnancy. Her only chronic medical condition was hypothyroidism that required no treatment. No family history of hereditary disease was known and BMI was in the normal range.

#### 2.2.2. Ultrasound Findings

The first trimester screening was performed in another clinic, NT was measured 3.2 mm and an agenesis of the DV was suspected; therefore, she was referred to our center.

We confirmed an NT of 3.4 mm, representing 99 percentile according to the CRL of 70.6 mm, a DV agenesis, an SUA, an EIF in the right ventricle and frontally depressed cranial bones. A follow-up ultrasound scan at the 16th gestational week confirmed these anomalies and the DV agenesis (intrahepatic portosystemic shunt). A fetal echocardiogram performed by a pediatric cardiologist showed no evidence of major cardiac malformation; however, a small subaortic ventricular septal defect (VSD) with a suspected septal membrane aneurysm was reported. At the 21st week, in addition to the previously described anomalies, an abnormal PFSR and heart ventricular hypertrophy were confirmed.

**Figure 4:**
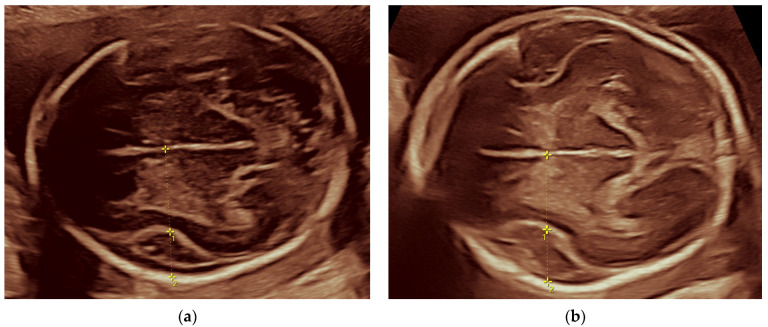
Measurements of the Sylvian fissure. Measurements marked with 1 indicate the insular depth, measurements marked with 2 indicate the Sylvian fissure depth. (**a**) The depth of the Sylvian fissure was measured 8.0 mm at the 19th week; (**b**) and 10.9 mm at the 22nd week. Both above the 95th percentile according to the database published by Alonso et al. in 2010 [16].

**Figure 5:**
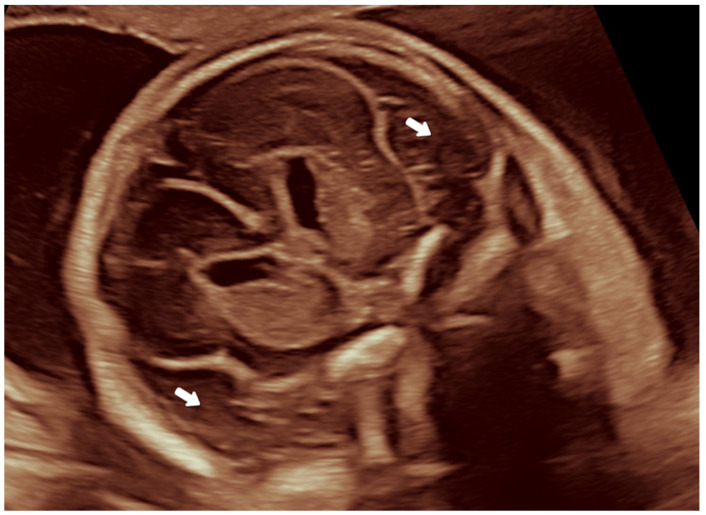
Coronal transcaudate plane in the 22nd gestational week. The dilation of the subarachnoid spaces and Sylvian fissures is notable (arrows).

At the 19th week, a dilated Sylvian fissure of 8.0 mm was found along with an otherwise normal neurosonography. At the 22nd gestational week, the depth of the Sylvian fissure was measured 10.8 mm (Figure 4). Both of these measurements represent values above the 95th percentile. The expansion of the subarachnoid spaces and Sylvian fissures was also remarkable in the coronal plane (Figure 5).

#### 2.2.3. Genetic Studies

No cf-DNA test was performed.

An amniocentesis was performed, and the results of the karyotyping and the SNP microarray revealed no pathologic findings. The whole exome sequencing found a heterozygous variation in the *PTPN11* gene (c.124A>G, p.Thr42Ala; chr12-112884189 A>G, NM_002834.5, rs397507501), confirming Noonan syndrome 1 (Phenotype MIM number: 163950, Location 12q24.13, Gene/Locus MIM number 1768762.2.4).

#### 2.2.4. Perinatal and Postnatal Outcome

Following detailed genetic counseling and receiving comprehensive information, the pregnant woman decided to terminate her pregnancy.

## 3. Discussion

NS is a common genetic disorder, with a high variability in both genotype and phenotype. Due to the diversity of the syndrome, prenatal and even postnatal detection is often challenging [17].

Although NS can be detected by various genetic testing modalities such as targeted Noonan panels, WES and even cf-DNA tests, screening tests are not available routinely in all countries and might be a significant financial burden for patients, while diagnostic tests are carried out only based on a proper indication. Therefore, sonographic markers that could raise suspicion of this condition are playing an important role in providing effective prenatal screening and improving the detection rate [17].

Noonan syndrome is suspected mainly due to increased NT in the first trimester [9,11]. However, access to first trimester screenings might be influenced by the patient’s socioeconomic status and personal preferences [18,19,20]. In a review published in 2024, Tangshewinsirikul et al. found increased NT to be the most frequently detected prenatal ultrasound anomaly in 105 cases of Noonan syndrome, occurring in 71% of the cases, followed by various body fluid collections (59%) and polyhydramnios (50%) [21]. Although in a study of 151 children with NS, only 12.5% of the echocardiograms were normal and 62% of the abnormalities were pulmonary stenosis. In the 105 fetuses with NS, the most commonly reported ventricular hypertrophy was found in 33% of the fetuses and pulmonary stenosis in only 13% [21,22].

The intracranial manifestations of NS have been described mainly in children or young adults and include hydrocephalus, Chiari I malformation, diverticular enlargement of the foramen of Luschka and cerebrovascular aneurysm [23,24,25]. Helenius et al. reported a 22-week-old fetus with enlarged extracerebral CSF and delayed operculisation in addition to a hypoplastic vermis on prenatal MRI in a case of NS with *RAF1* variation [26]. A widening of the subarachnoid spaces was described by Gripp in children with *SHOC2* and Zarate in a case with the *RAF1* variants, along with other extra- and intracranial abnormalities in individuals affected by NS. Mastromoro et al. described an MRI diagnosis of EH in the 27th gestational week following the genetic diagnosis of NS caused by a variation in the *PTPN11* gene. In their case, the corpus callosum and the cerebellar vermis were measured under the 10th percentile [27].

The prenatal MRI diagnosis of EH has previously been described in some disorders such as Snijders Blok–Campeau Syndrome and benign external hydrocephalus [28,29]. Baron et al. reported 21 cases of macrocephaly diagnosed by ultrasound, all of which had a fetal MRI, which confirmed the presence of EH in 77% of the cases [30]. In postnatal life, the enlargement of the subarachnoid fluid spaces was reported in connection with RASopathies [31].

A postnatally described central nervous system anomaly in the presence of the *SOS1* gene variation was a corpus callosum agenesis with severe developmental delay [32]. Another case report described spinal lesions resembling plexiform neurofibromas in NS caused by a variation in the *SOS1* gene [33].

The involvement of the two gene variants we identified, *SOS1* and *PTPN11*, in the development of the central nervous system has been previously reported. The *PTPN11* gene encodes the Shp2 protein and in NS it has a gain-of-function variation. In mice, Shp2 reduces the axon myelination and increases the density of the neurons but decreases the astrocyte density within the forebrain and the hippocampus. *SOS1* stimulates the nerve growth factor, which is expressed in the cortex of the newborns, and activates the Ras-MAPK pathway NMDA receptors in the neonatal cortex [34,35]. Whether these genes play a role in the development of EH in NS, and if so, via what exact pathway, requires further research.

In our two cases, EH occurred in the second trimester without any other significant central nervous system (CNS) anomaly and was confirmed ultrasonographically by measuring the depth of the Sylvian fissure in the axial plane. To our present knowledge, Case 1 is the first case of external hydrocephalus with an otherwise normal CNS in the presence of the *SOS1* gene variation. Furthermore, these are the first cases of NS with a second trimester ultrasound diagnosis of EH.

Neurosonography and MRI are complementary modalities in fetal diagnostics. According to the ISUOG guidelines, a fetal MRI is indicated in 7–15% of cases following a well-performed neurosonography [36]. In our experience, ultrasound examination alone was adequate for evaluating the subarachnoid spaces, offering better cost-effectiveness, faster diagnosis and lower human resource requirements compared to MRI. Despite the two cases presented, it must also be taken into account that EH may have developed independently of NS in both cases. However, since external hydrocephalus is a known phenomenon in children suffering from NS, it is reasonable to assume that the prenatal finding is related to the syndrome [26,37,38].

Examination of the subarachnoid spaces is currently not part of routine screening, and this may presumably contribute to undetected cases of external hydrocephalus. In the authors’ opinion, the measurement of the depth of the Sylvian fissure may be a suitable tool for a second trimester prenatal diagnosis of external hydrocephalus, and as an additional marker, could contribute to the timely implementation of genetic testing, which can be beneficial in some regions due to the legal regulation of termination. The application of Sylvian fissure depth measurement in this context requires further studies, but as its technical performance does not require the acquisition of new skills, it could be easily integrated into routine screening.

## Data Availability

The data presented in this study are available on request from the corresponding author due to privacy reasons.

## Abbreviations

The following abbreviations are used in this manuscript:

AC	Abdominal circumference
BMI	Body mass index
BPD	Biparietal diameter
cf-DNA	Cell-free DNA
CNS	Central nervous system
CRL	Crown-rump length
DV	Ductus venosus
DVP	Deepest (maximal) vertical pocket
EH	External hydrocephalus
EIF	Echogenic intracardiac focus
HC	Head circumference
IVF	In vitro fertilization
NS	Noonan syndrome
NT	Nuchal translucency
PFSR	Prefrontal space ratio
SUA	Single umbilical artery
VSD	Ventricular septal defect
